# Compact structured light generation based on meta-hologram PCSEL integration

**DOI:** 10.1186/s11671-023-03866-w

**Published:** 2023-06-19

**Authors:** Wen-Cheng Hsu, Chia-Hsun Chang, Yu-Heng Hong, Hao-Chung Kuo, Yao-Wei Huang

**Affiliations:** 1 Department of Photonics, College of Electrical and Computer Engineering, National Yang Ming Chiao Tung University, Hsinchu, 30010 Taiwan; 2 Semiconductor Research Center, Hon Hai Research Institute, Taipei, 11492 Taiwan

**Keywords:** Metasurfaces, Photonic crystal surface-emitting laser, Hologram, Structured light

## Abstract

Metasurfaces, a catalog of optical components, offer numerous novel functions on demand. They have been integrated with vertical cavity surface-emitting lasers (VCSELs) in previous studies. However, the performance has been limited by the features of the VCSELs such as low output power and large divergence angle. Although the solution of the module of VCSEL array could solve these issues, the practical application is limited by extra lens and large size. In this study, we experimentally demonstrate reconstruction of a holographic images using a compact integration of a photonic crystal surface-emitting laser and metasurface holograms designed for structured light generation. This research showcases the flexible design capabilities of metasurfaces, high output power (on the order of milliwatts), and the ability to produce well-uniformed images with a wide field of view without the need for a collection lens, making it suitable for 3D imaging and sensing.

**Supplementary Information:**

The online version contains supplementary material available at 10.1186/s11671-023-03866-w.

## Introduction

Metasurfaces and applications have attracted much attention in recent years because they provide manipulation of light propagation behavior on demand and solve the bulky size issue of conventional optical components by using semiconductor manufacturing techniques [1–6]. Nowadays, small size and lightweight wearable devices are in rapid development, which needs integration of active device and passive component to meet the expected in the future.

The integration of metasurfaces and vertical cavity surface-emitting lasers (VCSELs) is one of high-profile cases, as it offers the ability to enable the development of compact, efficient, and flexible devices with a wide range of functionalities. Xie et al. and Wang et al. [7, 8] demonstrated the successful integration of metasurfaces and VCSELs in control of the laser beam profiles and holographic display technologies. However, beam profile from VCSELs is limited by smaller single-mode light-emitting area, lower output power (usually be arranged in array for larger power) in a single chip, larger divergence angle. In contrast, photonics crystal surface-emitting lasers (PCSELs) provide higher output power source in a single chip and with an ultra-low divergence angle, eliminating the need for extra collimation lenses [9–11]. In the meantime, PCSELs surpass the advantages of VCSELs, including symmetrical beam patterns, wafer-level testing, and stable processes. These properties make PCSELs the best candidate for integrating with metasurfaces and surface-emitting lasers, reducing the overall volume in optoelectronic systems, and allowing for self-alignment of the light path in the packaging process. One of the technologies mentioned above is the face ID functionality of the iPhone, where a 3D face reconstruction and feature recognition are performed by structured light projection [12–14], imaging, depth estimation [15–17], and 3D reconstruction [18]. In commercial face reconstruction techniques, the structured light projector is composed of a VCSEL array (for higher output power ~ 3 W), a collimating lens, a flip light guide, a diffuser, and a series of diffractive optic elements (DOEs) for structured light generation [19]. However, the optical complexity of commercial dots projector, including extra collimation lens, light path alignment issue, and large size, results in difficulty in integration with light source by present semiconductor process.

**Fig. 1:**
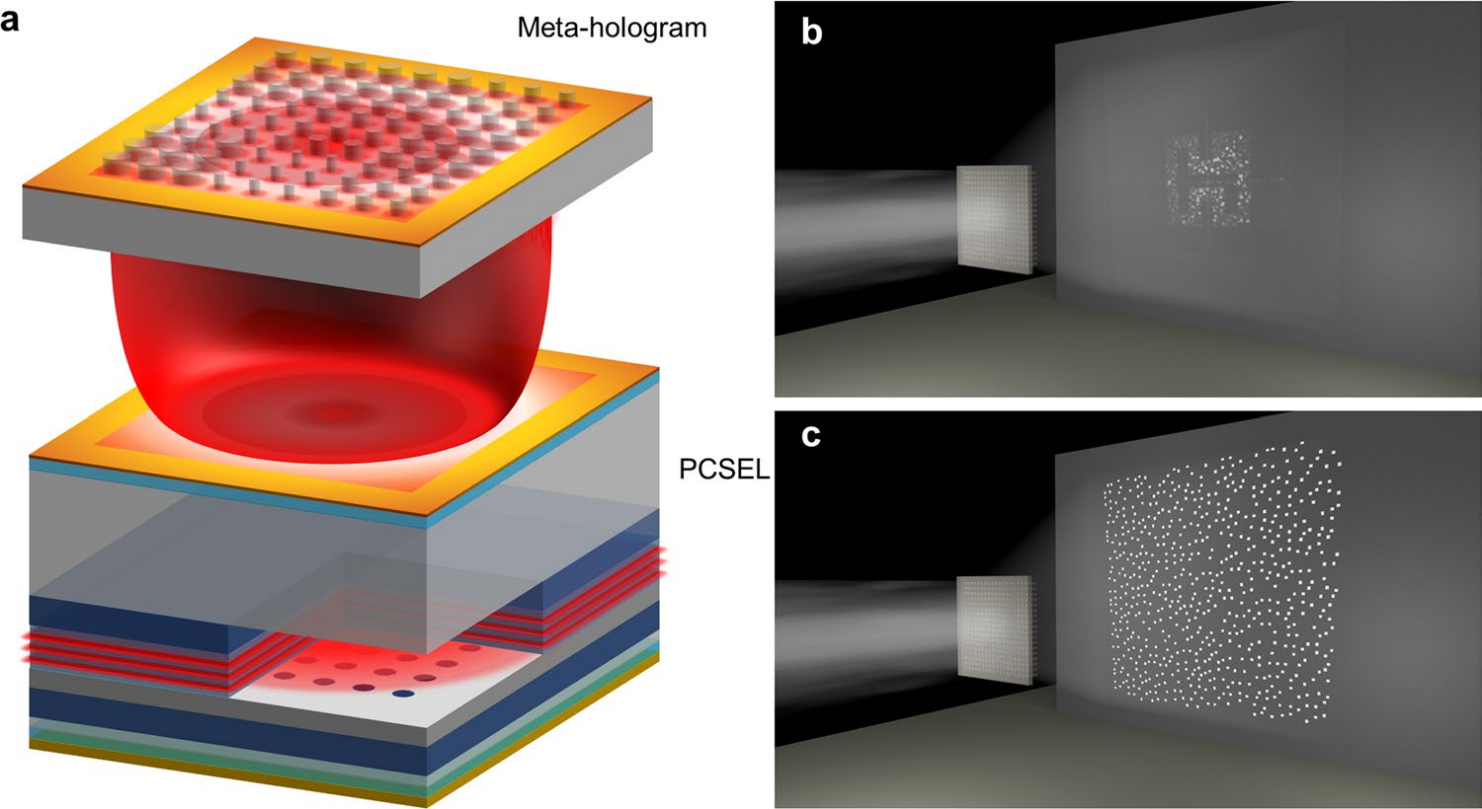
The illustration of metasurface, PCSEL, and desired holographic image. **a** Illustration of meta-hologram illuminated by photonic crystal surface-emitting laser, the laser spot generated by photonics crystal cavity and pass through the metasurface. The metasurface designed by GS algorithm acting as phase mask and project the desired holographic image. **b** Holographic image of Hon Hai logo projected from meta-hologram (sample 1). **c** Desired random beam profile for 3D face reconfiguration projected from meta-hologram (sample 4)

In this article, we demonstrate meta-pattern-projectors (MPPs) that integrated meta-optical element and optoelectronics. Our MPPs utilize meta-holograms and a PCSEL to reconstruct images with superior performance. The architecture of MPPs is depicted in Fig. 1a. We replace VCSEL array with single commercial PCSEL (L13395-04, Hamamatsu) for higher output power in a smaller area. Collimation lenses or additional lens phase profile is not necessary in the optical system because of the ultra-small divergence angle (< 1°) beam profile output from PCSEL. The hologram design in metasurface replaces DOEs for pattern projecting and device miniaturization, the subwavelength pixel size in metasurface also improves the efficiency, especially at large diffraction angle [20–23]. Our scheme provides a compact form, dramatically reducing the optical complexity of the system. Figure 1b, c shows schematic of structure light generation from our MPP samples, including holographic images of the Hon Hai logo (sample 1 without padding and sample 2 with padding) and random dot patterns (sample 3 and sample 4), respectively. Detailed specification of meta-hologram samples is listed in Additional file 1: Table S1. To eliminate high-order diffraction patterns within the observable diffraction angle, padding (blank area) is applied in the ideal image (around the Hon Hai logo or random dot patterns). Overall, our MPPs provide potential applications in future consumer electronics, including barcode scanning, facial recognition, and other areas that require high-quality image reconstruction. The combination of meta-holograms and PCSELs also leads to further advancements in optoelectronics and holographic technologies.

## Design of meta-hologram

Commercial 940-nm PCSEL is used as light source. Photonic crystal composed of asymmetric geometry hole is design for high output power, accompanying with small values (~ 0.534 in our case, Additional file 1: Fig. S1a) of degree of polarization (DOP). Compared to general PCSEL, high-power PCSEL breaks the symmetry in a unit cell and reduces destructive interference in the far field, and these lead to raising up of the threshold current and DOP. Considering the polarization feature of PCSEL, we design polarization-insensitive metasurface hologram with circular and square nanopillars.

To design the meta-hologram, the first step is to build a library of meta-atoms with 2π phase coverage and high transmission using the rigorous coupled wave analysis (RCWA) method [24]. The lattice constant is selected based on the Nyquist sampling criterion (lattice < *λ*/2*NA*), which requires a lattice constant less than 470 nm considering the operation wavelength of 940 nm. In this study, an 800-nm-height circular (sample 1–3) or square (sample 4) pillar in a square lattice with a lattice constant of 330 nm was selected after optimizing in regard of higher transmission efficiency. The detailed meta-atom library is shown in Additional file 1: Fig. S2c, where transmittance of selected pillars is between 65 and 85%. The meta-hologram is composed of GaAs pillars directly etched from a GaAs substrate, with a refractive index of 3.56 at 940 nm. This substrate can be replaced by n-type GaAs substrate of a flip-chip PCSEL for avoiding the refractive index mismatch and improving the transmission efficiency in the system.

In order to obtain the desired holographic image, the iterative algorithms based on Fourier integral are employed which is widely used for DOEs and computer-generated holograms. One of the most used is Gerchberg–Saxton (GS) algorithm, which is employed to obtain the desired intensity distribution at an observation plane placed at the far field or at the focal plane of a lens [25, 26]. Here, we use fast Fourier transform and inverse fast Fourier transform in the kernel of algorithm, which represents the Fraunhofer propagation because its characteristic is suitable in long-distance application. The iterated error is defined as difference between each numerical image and desired image. The iterated error curve and numerical results are shown in Additional file 1: Fig. S2.

**Fig. 2:**
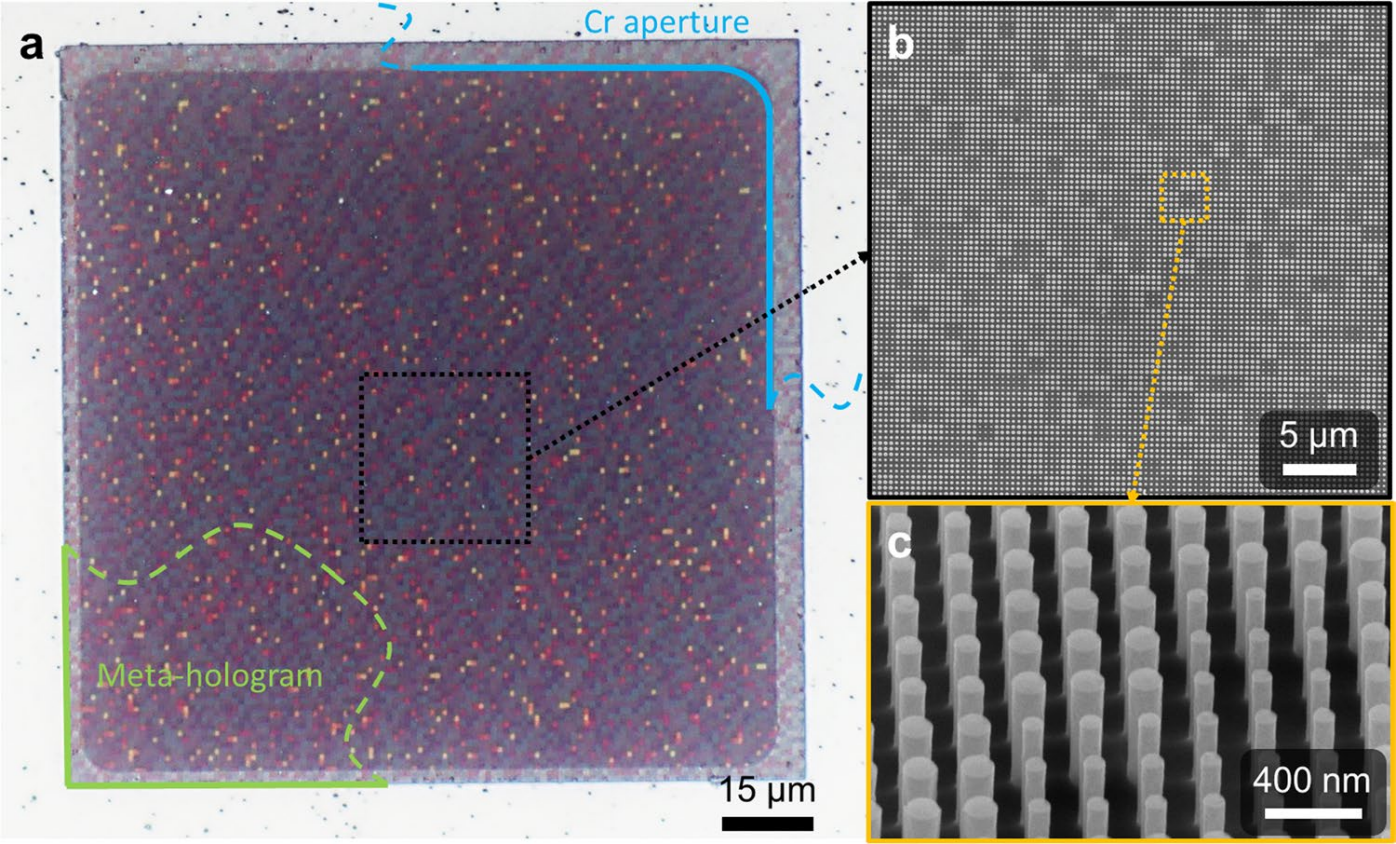
Fabricated meta-hologram sample. **a** Optical microscopic (OM) image of metasurface (top view) with the full size of ~ 119^2^ μm^2^. The meta-hologram consists of 120^2^ pixels. Each pixel is composed with 3 × 3 meta-atoms, with lattice constant of 330 nm. A 200-nm-thick Cr aperture is made around the meta-hologram. **b** Scanning electron microscopic (SEM) images of sample 1. **c** The tilted and closed-up SEM image of sample 1

The meta-hologram (sample 1) in this design is sampling as 120 × 120 pixels, and each pixel size is 990 nm and composed with a 3 × 3 meta-atom array. The difference in colors in each pixel comes from the difference width of the pillars. The small pixel size makes the metasurface more efficient than conventional DOEs or spatial light modulator (SLM), which have pixel sizes on the order of microns. The pixel size and numbers of pixels in the meta-hologram determine the accuracy of the generated image. Each pixel induces a diffraction grating effect and generates high-order diffraction images in the corresponding diffraction angle. According to the diffraction formula: $\theta_{d}^{m} = \sin^{ - 1} m\lambda /n\Lambda$ (*m* is the diffraction order, *λ* is the working wavelength, *n* is the super-lattice number, and Λ is the period of the meta-atom), the 1st diffraction angle reaches 71°. The large diffraction angle of the meta-hologram results 84° azimuthal angle in desired pattern, which is confirmed by FDTD simulation. The optical microscopic (OM) and scanning electron microscopic (SEM) images in Fig. 2 show the 3 × 3 pillars composing a pixel, with a pillar height estimated at about 800 nm and a vertical side at about 90°, indicating the circular columnar structure produced by the GaAs etching process is nearly perfect. The area around the metasurface is covered by 200-nm-thick Cr as aperture shown in Fig. 2a.

## Fabrication of meta-holograms

We deposit a near 100-nm-thick Si_3_N_4_ layer at 300 °C using plasma-enhanced chemical vapor deposition (Plasmalab80Plus, Oxford Instruments) on a 360-μm-thick GaAs substrate as a hard mask, followed by spin-coating a negative electron-beam resist (ma-N 2403, Micro Resist Technology). The meta-hologram pattern is defined by using electron-beam lithography (ELS-7500, Elionix) with current 300 pA and 50 keV, followed by nanostructure dry etching on the Si_3_N_4_ layer using inductively coupled plasma (Plasmalab System 100, Oxford Instruments). The pattern is then transferred from the Si_3_N_4_ hard mask to the GaAs substrate using a mixture of Ar_2_ and SiCl_4_. The residual Si_3_N_4_ was removed by dipping in buffer oxide etch (BOE) for 1 min. We fabricate a metal aperture after the metasurface fabrication process. The aperture pattern is defined by using ultraviolet lithography, followed by depositing 200-nm-thick Cr using a thermal evaporator and lift-off process.

## Results and discussion

### Optical characteristics of PCSEL

The threshold current of the commercial PCSEL is around 200 mA with a slope efficiency of 0.495 W/A, and an output power of around 36 mW at 300 mA. The features of commercial PCSEL are measured and shown in Additional file 1: Fig. S1, including DOP, the detailed light power, current and voltage (LIV) curve, spectrum, far field, and beam profile projected onto sample operating at room temperature. The center wavelength of PCSEL is around 940.2 nm with a FWHM of 0.02 nm under operation current 300 mA (Additional file 1: Fig. S1c and S1d), and it has a small FWHM divergence angle of around 0.300°, as shown in the far-field measurement in Additional file 1: Fig. S1e. Compared to VCSELs, PCSEL performs smaller divergence angle, resulting in no additional collimator phase profile in the metasurface. Therefore, it is easier and more convenient to integrate metasurface and PCSEL because of no optical axis of collimator to be aligned.

### Characteristics of meta-hologram

**Fig. 3:**
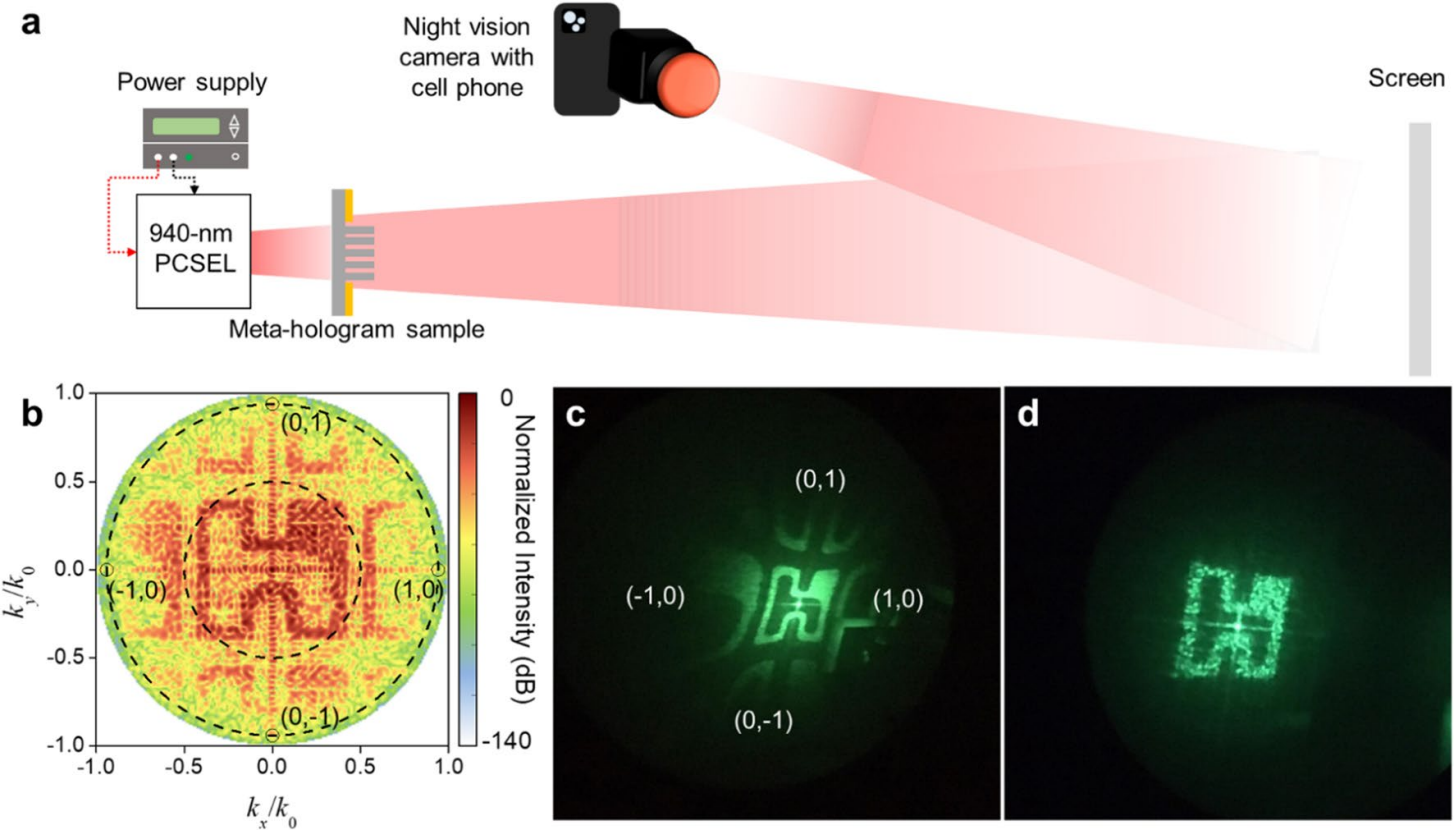
Experimental setup of meta-hologram and reconstructed holographic images in simulation and experiment. **a** Illustration of measurement setup. A 940-nm PCSEL is used as light source and illuminated on metasurface sample. The sample projects desired holographic image on the screen and image is recorded by night version gaggle and smartphone camera. **b** Numerically reconstructed image of Hon Hai logo, which the main logo is designed within ± 30° (inner dashed line). First diffraction orders are designed at ~ 70° (marked as (1, 0), (− 1, 0), (0, 1), and (0, − 1) on outer dashed line), accompanying part of 1st diffraction patterns (between inner and outer dashed line). **c** Measured image of Hon Hai logo projected from meta-hologram sample 1. **d** Measured image of Hon Hai logo with image padding process projected from meta-hologram sample 2

To predict and verify the reconstructed holographic images of sample 1, we generate a smaller metasurface pattern (30 × 30 pixels, 29.7^2^ μm^2^) and import it into Lumerical FDTD for far-field calculation. The light source is 940 nm plan wave, and simulation results are shown in Fig. 3b. The intensity of red area is 100–1000 times larger than yellow area, indicating high contrast between reconstructed image and background noise.

The experimental setup of holographic reconstructed image is shown in Fig. 3a. A commercial 940-nm PCSEL as laser light source with operation current of 300 mA. A meta-hologram sample is fixed on a 3-axis stage. The distance between PCSEL and sample is ~ 10 mm, where the laser spot size on sample is ~ 277 μm. The holographic images then project onto a flat screen 15 cm away from meta-holograms, which satisfies the Fraunhofer diffractive condition.

In the experiment, the ideal holographic image of Hon Hai logo is designed with 120 × 120 pixels. The overall size of fabricated meta-hologram sample 1 is ~ 119^2^ μm^2^. Figure 3c shows an experiment image, which is clearly distinguishable and matches well with the simulated result shown in Fig. 3b. The central light spot is the 0th diffraction. High-order diffractions of spots and images of (1, 0), (− 1, 0), (0, 1), and (0, − 1) can be observed beside the main image.

To eliminate the high-order diffractions images, padding process is utilized by filling with zeros around the ideal image [27]. The original ideal image is 30 × 30 pixels and extended to 100 × 100 pixels after padding process. The overall size of fabricated meta-hologram sample 2 is 99^2^ μm^2^. The reconstructed image of Hon Hai logo with padding process is shown in Fig. 3d. High-order diffraction images are eliminated.

We measure the efficiency of the meta-hologram sample 2 because of its pure 0th-order diffraction images. The relative efficiency is defined as:

1 $$E_{r} = \frac{{P_{m} - P_{0} }}{{P_{{{\text{GaAs}}}} }},{ }$$

where *P*_*m*_ is received power of the 0th-order image and spot after laser pass through a meta-hologram, *P*_*0*_ is the power of the 0th-order spot, and *P*_GaAs_ is received power after laser pass through the GaAs substrate. The size of meta-hologram is smaller than the beam size of the laser. Therefore, we utilize two metal apertures (with and without sample) fabricated on the substrate for efficiency measurement. To measure the absolute efficiency, the input power from PCSEL (with a same-sized aperture) can be estimated considering transmittance of air–GaAs boundary twice to the *P*_GaAs_, which is *P*_GaAs_/46.9%. Therefore, the absolute efficiency *E*_*a*_ is determined as *E*_*r*_ × 46.9%. As results, relative efficiency of up to 54.8% and absolute efficiency of 25.7% are measured, considering samples with different exposure times in the electron-beam lithography process (Additional file 1: Table S1).

**Fig. 4:**
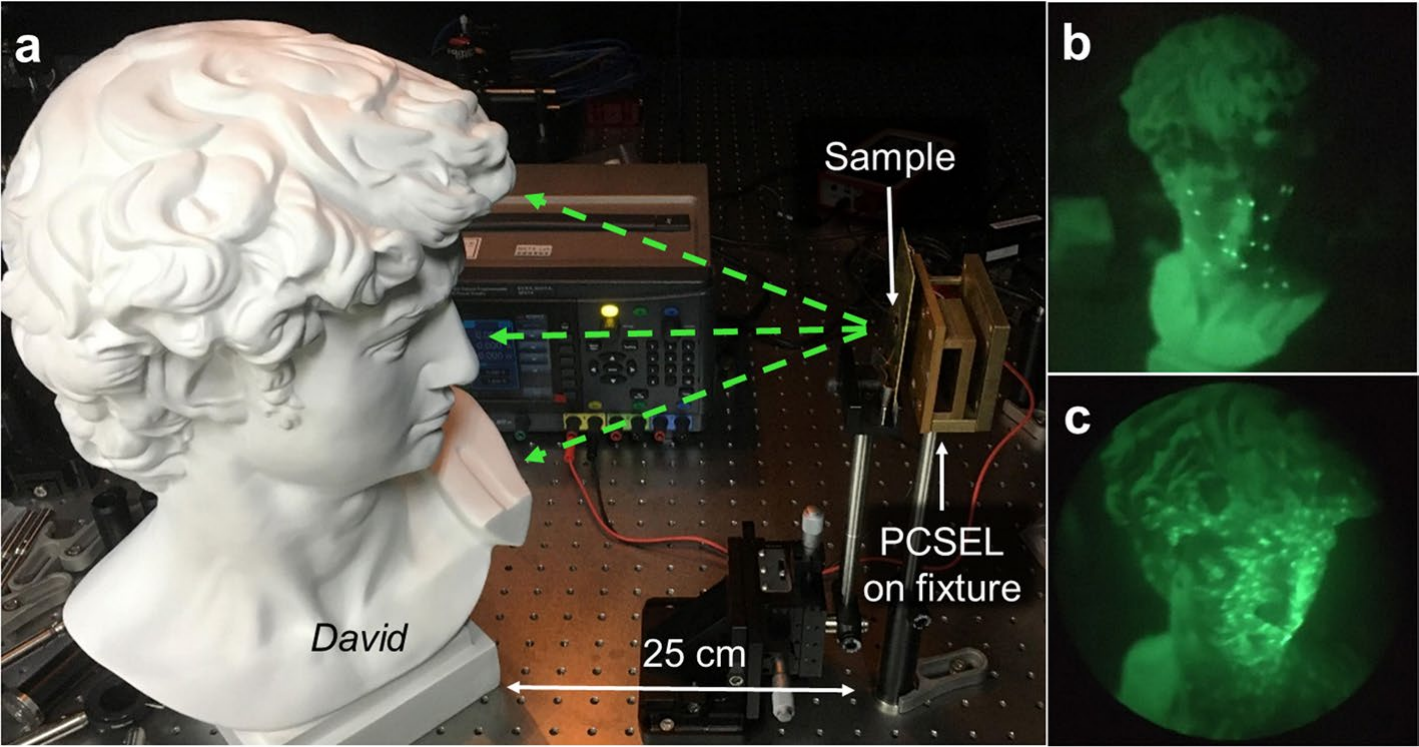
Comparison of random beam structured light generated by meta-hologram. **a** Demonstration of multi-beams projection on the statue of *David*. The distance between *David* and metasurface sample is about 25–50 cm. **b** Holographic image of 31 spots random beam from sample 3 projected on *David*. **c** Holographic image of 750 spots random beam with Gaussian distribution spots from sample 4 projected on *David*

We also demonstrate random dots pattern for future 3D depth sensor based on structured light system. The design rules of structured light image are based on the purpose of facial recognition, as shown in Fig. 4a. The detection distance between face and our meta-hologram is from 25 to 100 cm, and the length of face is about 30 cm. Therefore, the requirements result in FOV of larger than 62°. We design and demonstrate two samples (meta-hologram sample 3 and sample 4) with different number of light dots and shape of spot. And we check if the density and brightness of dots are enough. Figure 4b and 4c shows reconstruction holographic images of random dots projected onto statue of *David’s* face. (The size of *David* is 30 cm × 20 cm × 11 cm.) In Fig. 4b, 31 spots in Dirac-delta distribution are demonstrated and projected on *David*. On the other hand, 750 spots in Gaussian distributions are demonstrated and shown in Fig. 4c. Both two spots design strategies illuminate the object and cover full surface of target located at 25–50 cm away. Results indicate that the spot design in Dirac-delta distribution is more bright and higher contrast than that design in Gaussian distributions.

The system works in free space environment at room temperature, and making the metasurface design combined with a PCSEL is highly suitable for structured light-based depth-sensing applications. Shpunt et al. [28] first utilized structured light technology for motion-sensing game projecting lots number of tiny dots from a projector to sense the posture. Pesach et al. [29] miniaturized this technology and compacted it into cell phone for face recognition, which projects over 32 K dots onto human’s face and reconstructs the 3D face model for eigenface data extraction. The thousands of dots are projected from a dot projector composed with a VCSEL array and a compactable optical module that includes a collimation lens, light guide, and serious of DOE. The VCSEL array provides spread of laser light, gathered by the collimation lens and injected into DOE, splitting a light spot as thousands of dots. Dot projection from integration of bulky laser source (e.g., supercontinuum laser, tunable laser, He–Ne laser, etc.) with metasurface is perfectly performance in previous articles but failed to compact in wearable devices [30–34]. In our MPP, the DOE and VCSEL array are replaced by our meta-hologram device and PCSEL for smaller form factor. This scheme requires no collimator and demonstrates the function of dots projector in more simple architecture for system compactable.

## Conclusion

To summarize, we used the GS algorithm to design the Hon Hai logo under the Fraunhofer condition and reconstructed the holographic image on an object 25–50 cm away, illuminated with a commercial PCSEL operating at wavelength of around 940 nm. A clear and well-uniform experimental holographic image was observed. The FOV of our meta-hologram is 84° and can be even higher in theory. Afterward, we show that this device has great potential in structured light 3D sensing by demonstrating the dots projector system in Fig. 4. The scale size was reduced from the module level to a single laser diode level, and the complexity of the optical module can be largely reduced. The output power of a PCSEL is much greater than a VCSEL, and it makes our MPP have enough working distance and can be applied in realistic. The mono-integration can be further realized through the flip-chip PCSEL fabrication process, which compacts the meta-hologram structure onto the GaAs substrate of PCSEL. This method avoids refractive index mismatch issue, reduces the complexity of optical system, and improves the transmission efficiency ~ 2 times in the whole optoelectronics system. The light path between metasurface and PCSEL would do self-alignment in fabrication process and the alignment issue in bonding or package process also be solved. Besides the silicon photonics, the architecture of the metasurface and laser device we demonstrated allows for a compact optoelectronic system and opens the path for miniaturized in wearable devices, such as depth sensors in sweeping robots and facial recognition functionality in mobile phones, augmented reality, and virtual reality systems [35–37].

## Methods

The setup of efficiency measurement starts from light path alignment from source through sample and iris to power meter. We record the output power of PCSEL without GaAs substrate by power meter. The GaAs substrate is then inserted between the light source and the power meter, and the output power passing through the substrate is recorded. Finally, the substrate is replaced by meta-hologram sample and reconstructed holographic image is projected into the area of power meter. The image intensity is recorded as the received power passing through the sample.

The size of projected image is smaller than the area of power meter, and this could be checked by IR card. The 0th spots extracted method is shown in Additional file 1: Fig. S3. To extract the spot intensity of 0th diffraction from holographic image, an iris is used to block the holographic image and allows the 0th spot passing through a central pinhole, and it makes only the 0th spot which is received by power meter.

## Supplementary Information

**Additional file 1**. Supplementary figures and Tables.

## Data Availability

All data generated or analyzed during this study are included in this published article.
