# Supplementary material for: Compact structured light generation based on meta-hologram PCSEL integration
Source: Discov Nano. 2023 Jun 19;18(1):87. doi: 10.1186/s11671-023-03866-w (PMC10279613; doi:10.1186/s11671-023-03866-w)
Supplement: Supplementary file 1 — Additional file 1. Supplementary figures and Tables. [file 11671_2023_3866_MOESM1_ESM.docx]

Supplementary Material of

**Compact structured light generation based on meta-hologram PCSEL integration**

**Wen-Cheng Hsu,^1, 2^ Chia-Hsun Chang,^1^ Yu-Heng Hong,^2,*^ Hao-Chung Kuo,^1,2,*^ and Yao-Wei Huang^1,*^**

1. Department of Photonics, College of Electrical and Computer Engineering, National Yang Ming Chiao Tung University, Hsinchu 30010, Taiwan

*2. Semiconductor Research Center, Hon Hai Research Institute, Taipei 11492, Taiwan*

*Correspondence and requests for materials should be addressed to:

[enoch.yh.hong@foxconn.com](mailto:enoch.yh.hong@foxconn.com), [hckuo0206@nycu.edu.tw](mailto:hckuo0206@nycu.edu.tw), [ywh@nycu.edu.tw](mailto:ywh@nycu.edu.tw)


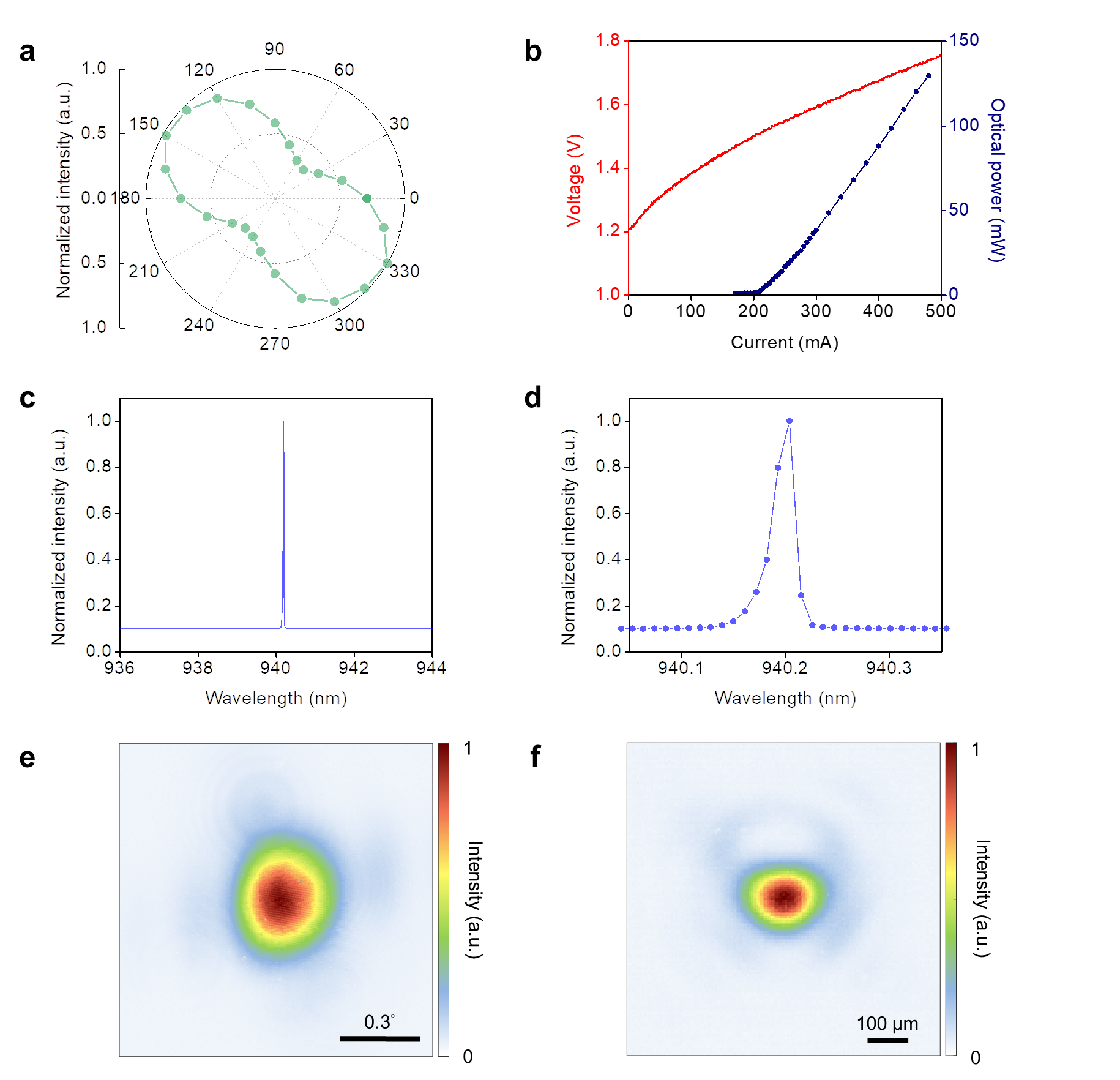


**Figure S1** **a** DOP of PCSEL measurement result at 300 mA. It represents the linear polarization feature and DOP is 0.534. **b** The light power, current and voltage (LIV) curve of the commercial PCSEL at room temperature. **c** The spectrum of the PCSEL biased at 300 mA in room temperature, indicating its single mode profile. **d** Magnified spectrum of the PCSEL from b, indicating that the center wavelength is at 940.2 nm. **e** The far-field profile of PCSEL captured by using a beam profilometer (Beamage-4M, Gentec-EO), the measurement distance between PCSEL and sample is 5.5 cm, the divergence angle can be estimated about 0.300^o^ under 526 mA. **f** The beam profile of PCSEL projected onto meta-holograms captured by using beam profilometer ~ 1cm away from PCSEL operating at current of 300 mA.


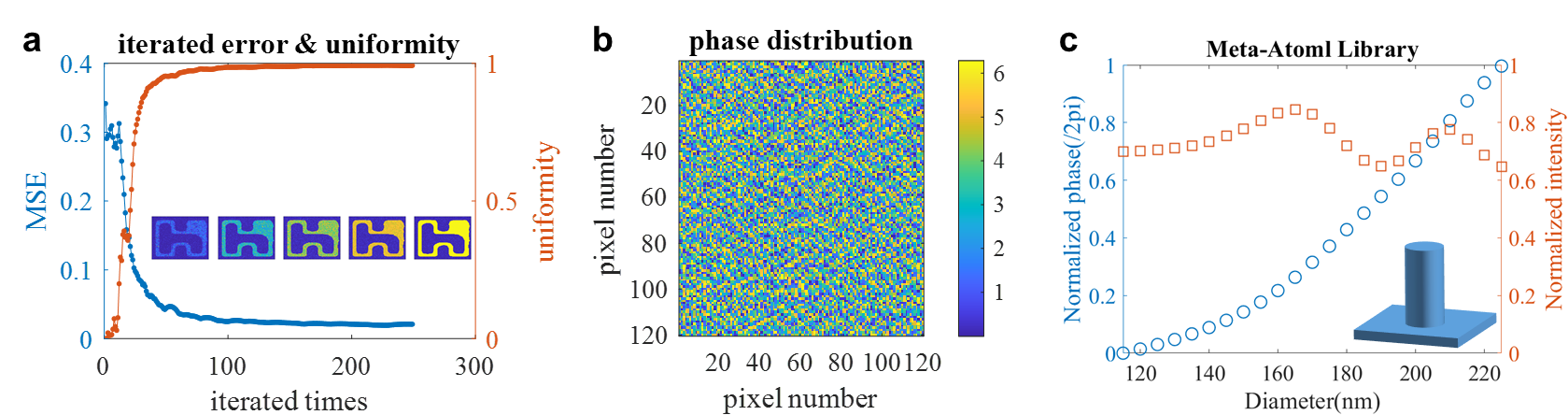


**Figure S2** **a** The iteration curve of MSE and uniformity in GS algorithm, blue curve is MSE variation, orange is uniformity in signal domain. The insert image is numerical result after 5, 15, 25, 50, 250 iterated time. Hon Hai logo is sampling to 120 $\times$ 120 pixels and calculated reconstruction image intensity by GS algorithm, which MSE and uniformity is convergence and image intensity variation dependent on iterated times. **b** The requirement of phase distribution when error of reconstruction image is less than criteria, scale bar is from 0 to 2π. **c** The meta-atom library calculated by RCWA, blue circle mark curve is normalized phase and orange square mark curve is normalized intensity.


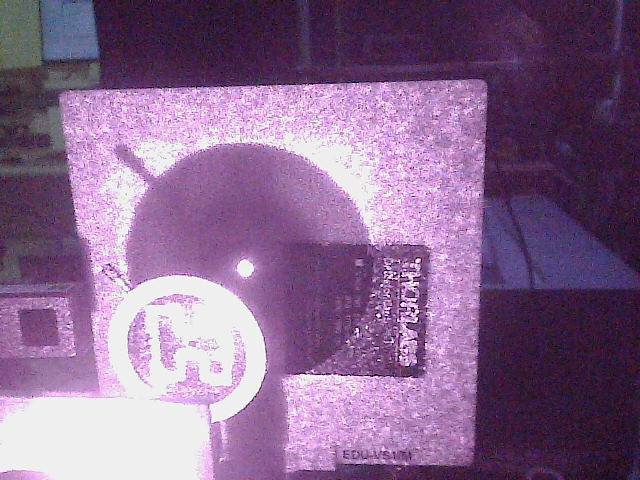


**Figure S3** The intensity of zero order extracted method by iris filter and used webcam to check the IR light path. The reconstruction holographic image projection into iris, only zero order spot pass through the pin hole in the center of iris, the intensity of detection size in power meter is smaller than iris.

| Sample | Pattern | Pixel number | Size (μm^2^) | Type of meta-atoms |
| --- | --- | --- | --- | --- |
| 1 | Hon Hai logo w/o padding | 120 × 120 | 119 × 119 | Circular pillars |
| 2 | Hon Hai logo w/ padding | 100 × 100 | 99 × 99 | Circular pillars |
| 3 | 31 spots random beam in Dirac-delta distribution | 100 × 100 | 99 × 99 | Circular pillars |
| 4 | 750 spots random beam in Gaussian distributions | 300 × 300 | 297 × 297 | Square pillars |

**Table S1** Specifications of meta-hologram samples.

|  | Sample 2a | Sample 2b | Sample 2c | Sample 2d | Sample 2e | Sample 2f |
| --- | --- | --- | --- | --- | --- | --- |
| Dose time (μs) | 0.28 | 0.29 | 0.3 | 0.31 | 0.32 | 0.33 |
| Power of all diffraction, *P_m_* (mW) | 7.71 | 7.52 | 7.72 | 7.49 | 7.52 | 7.21 |
| Power of 0^th^ order, *P*_0_ (mW) | 0.03 | 0.03 | 0.05 | 0.03 | 0.04 | 0.07 |
| Power of hologram image, *P_m_*-*P*_0_ (mW) | 7.68 | 7.49 | 7.67 | 7.46 | 7.48 | 7.14 |
| Input power (mW) | 32.3 | 32.3 | 32.3 | 32.3 | 32.3 | 32.3 |
| Power of GaAs substrate w/ aperture, *P_GaAs_* (mW) | 14.62 | 14.62 | 14.62 | 14.62 | 14.62 | 14.62 |
| Relative efficiency, *E_r_* | 52.5% | 51.2% | 52.5% | 51% | 51.1% | 48.8% |
| Absolute efficiency, *E_a_* | 23.8% | 23.2% | 23.7% | 23.1% | 23.2% | 22.1% |

**Table S2. Efficiency measurement result of sample 2 with different fabrication tolerance.** Samples 2a-2f were fabricated by using electron beam lithography with different dose time. Relative efficiency and absolute efficiency are defined in the main text.
